# Role of Oxide/Metal Bilayer Electrodes in Solution Processed Organic Field Effect Transistors

**DOI:** 10.1038/s41598-019-43237-z

**Published:** 2019-04-30

**Authors:** Abduleziz Ablat, Adrica Kyndiah, Geoffroy Houin, Tugbahan Yilmaz Alic, Lionel Hirsch, Mamatimin Abbas

**Affiliations:** 1 CNRS, Université Bordeaux, Laboratoire de l’Intégration du Matériau au Système (IMS), UMR 5218, ENSCBP, 16 avenue Pey Berland, 33607 Pessac Cedex, France; 2 School of Physical Science and Technology, Xinjiang University, Urumqi, 830046 People’s Republic of China; 3 Advanced Technology Research and Application Center, Selcuk University 42031, Campus, Selcuklu, Konya, Turkey

**Keywords:** Electrical and electronic engineering, Electronic devices

## Abstract

High performance, air stable and solution-processed small molecule 2,7-dioctyl[1]benzothieno[3,2-b]benzothiophene (C_8_-BTBT) based organic field-effect transistors (OFETs) with various electrode configurations were studied in detail. The contact resistance of OFET devices with Ag, Au, WO_3_/Ag, MoO_3_/Ag, WO_3_/Au, and MoO_3_/Au were compared. Reduced contact resistance and consequently improved performance were observed in OFET devices with oxide interlayers compared to the devices with bare metal electrodes. The best oxide/metal combination was determined. The possible mechanisms for enhanced electrical properties were explained by favorable morphological and electronic structure of organic/metal oxide/metal interfaces.

## Introduction

Organic field effect transistors (OFETs) have been attracting a major attention in scientific community of organic semiconductors, due to their bright future as building blocks of flexible electronic devices^1–3^. Since the first report by Tsumara *et al*.^4^, the performance and design of OFETs have been improved significantly and considered to be advantageous over the traditional amorphous silicon based field effect transistors^5,6^, which witnessed an increase from the first reported mobility value of 10^−5^ cm^2^/Vs^4^ to 43 cm^2^/Vs^7,8^ in newly emerged symmetric small molecule C_8_-BTBT (2,7-dioctyl [1]benzothieno[3,2-b]benzothiophene)^9^. Such an impressive development is due to rational chemical design, as well as better nano-morphology, crystallinity and quality of the films prepared by various growth methods^3,10,11^. Interfaces both at electrode/organic layer and dielectric/organic layer play significant role in the OFET device performance, where together with mobility, important parameters such as threshold voltage, subthreshold slope, hysteresis, current on/off ratio etc. become relevant in final device application. Electrode/organic layer interface mainly determine the contact resistance in OFETs, which becomes a main factor in limiting the device charge carrier mobility and switching speed, especially when the channel length is reduced. Contact resistance also affects strongly the threshold voltage of an OFET.

Therefore, in order to achieve high performance OFETs, the choice of appropriate electrodes has become the main research topic in recent literatures. In C_8_-BTBT based OFETs, Ag and Au have been commonly used as source/drain contacts. In the works of Yuan *et al*.^7^ and Minemawari *et al*.^8^, very high hole mobility OFETs were realized using Ag and Au electrodes, respectively. However, the reason for using either Au or Ag as an electrode was not discussed and compared in their studies. Other device parameters, notably threshold voltage were barely mentioned, which is understandable, since the focus of those studies was on the deposition techniques to enhance the mobility. Kano *et al*.^12^ reported C_8_-BTBT based OFETs using MoO_3_ as an interlayer between the Au electrode and C_8_-BTBT organic layer. The OFETs with oxide interlayer presented improved threshold voltage, subthreshold slope and strong suppression of the short-channel effect compared to those OFETs with Au electrode only. The improved device performance was attributed to the reduced charge injection barrier by MoO_3_ interfacial layer. However, to the best of our knowledge, no comparative study has been reported on the influence of various electrode/organic layer interface (metal/semiconductor and metal/metal oxide/semiconductor) on the properties of C_8_-BTBT based OFETs, which may guide one in choosing the proper source/drain electrode with corresponding interfacial layers for this important high performance small molecule.

In this work, we studied the effect of different metals and their combination with interfacial layers as electrodes for C_8_-BTBT based OFETs. We first separately fabricated devices with Au and Ag only electrodes and then compared with devices which incorporate MoO_3_ or WO_3_ interlayers. The best metal/oxide combination was identified and quantitative contact resistance analysis was carried out. Finally, we discussed the possible origin of the differences in the performance of OFETs by studying the energy levels of these interfaces with photoelectron spectroscopy and through the morphological analysis using atomic force microscopy (AFM).

## Results and Discussion

**Figure 1:**
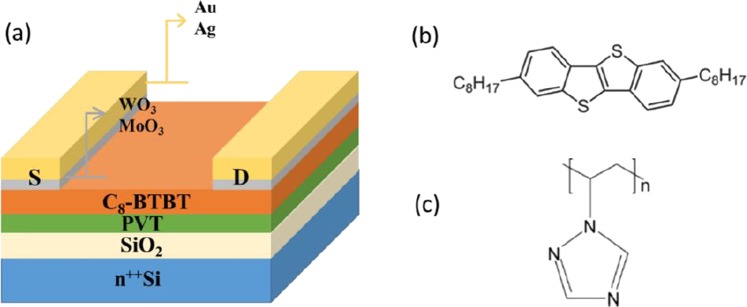
(**a**) Device structure of bottom gate-top contact (BG-TC) OFETs with different metals and interfacial layers; Chemical structures of C8-BTBT (**b**) and PVT (**c**).

**Figure 2:**
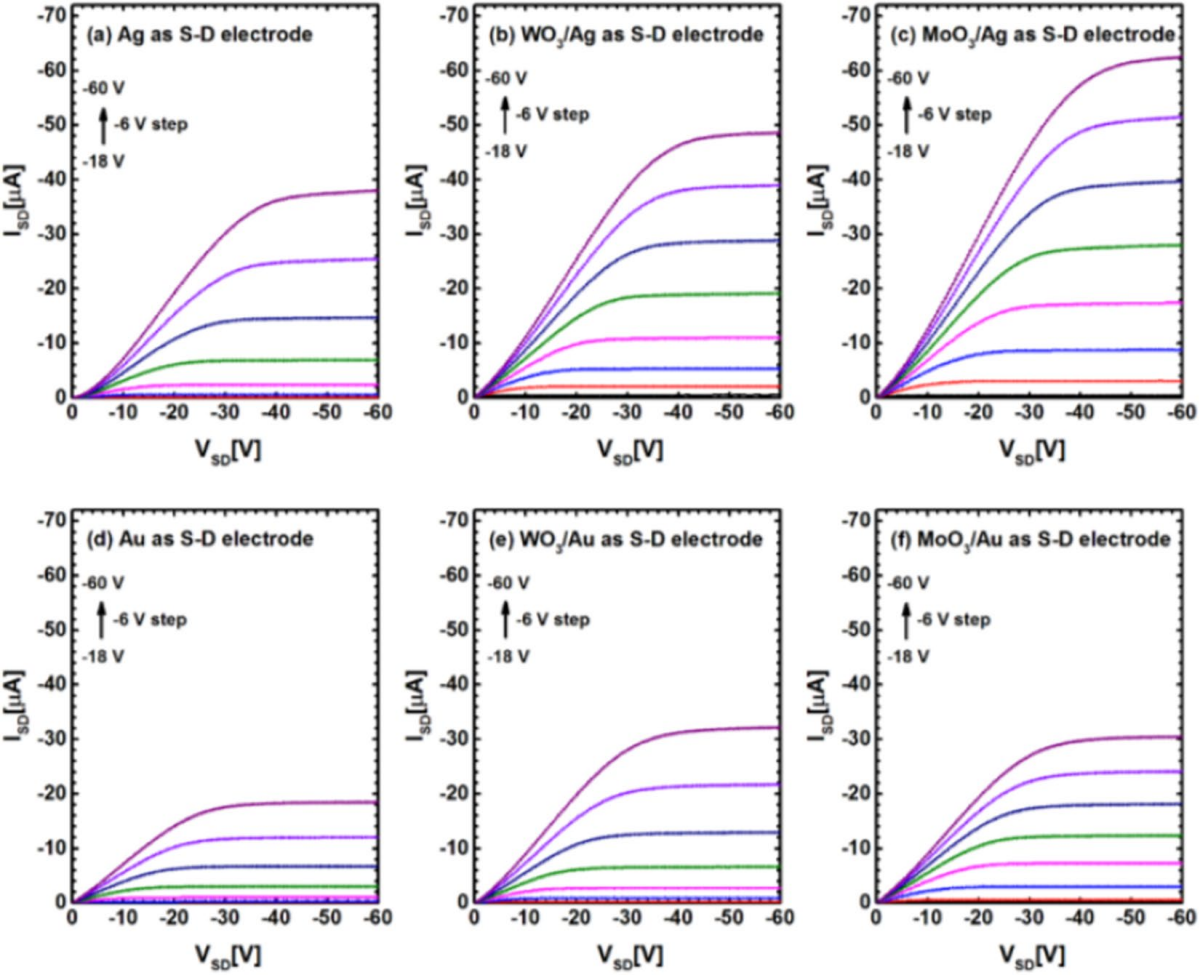
A comparison of output characteristics for various gate voltages (V_GS_) with different source/drain electrodes of OFETs: (**a**) bare Ag, (**b**) WO_3_/Ag, (**c**) MoO_3_/Ag, (**d**) bare Au, (**e**) WO_3_/Au and (**f**) MoO_3_/Au.

Bottom gate, top contact (BG-TC) OFET device structure is schematically shown in Fig. 1(a), where Au or Ag is served as source/drain electrode with or without MoO_3_ and WO_3_ interlayers. Chemical structures of active layer C_8_-BTBT and passivation layer poly (1-vinyl-1, 2, 4-triazole)^13^ are also presented (Fig. 1(b) and (c), respectively). The output characteristics mesured at various gate voltages (V_GS_) are shown in Fig. 2(a–f). All six I_SD_-V_SD_ plots of different source/drain electrodes with same channel length of 100 µm show excellent behaviors of drain current in linear and saturation regions, exhibiting typical transistor characteristics. However, obvious differences in device performances with different source/drain electrodes can easily be observed, especially in the linear region. When we compare the output curves of bare Ag and Au electrode OFETs (Fig. 2(a) and (d), respectively), the on-state current of Ag-based devices (−38 µA) is more than twice higher than that of Au-based devices (−18 µA) at the same V_SD_ and V_GS_ (V_SD_ = −60 V and V_GS_ = −60 V). That (This instead of That) may be the reason that the highest mobility was achieved with Ag rather than Au electrode for C_8_-BTBT based devices^7,8^. However, stronger deviation from linearity at low V_SD_ in Ag device suggests that Ag electrode renders higher contact resistance than Au electrode. After inserting the thin oxide layers such as WO_3_ and MoO_3_ between the metal electrode and active layer, the output current of the devices increased further comparing to the devices with bare metal electrodes (as shown in Fig. 2(b),(c),(e) and (f)), notably with improved linearity at low V_SD_, which indicates the decrease of the contact resistance in these devices. We note that the highest on-state current was obtained with MoO_3_/Ag electrode. We further compared other parameters among the devices in order to figure out the general trend in OFET performances with different source/drain electrodes.

**Figure 3:**
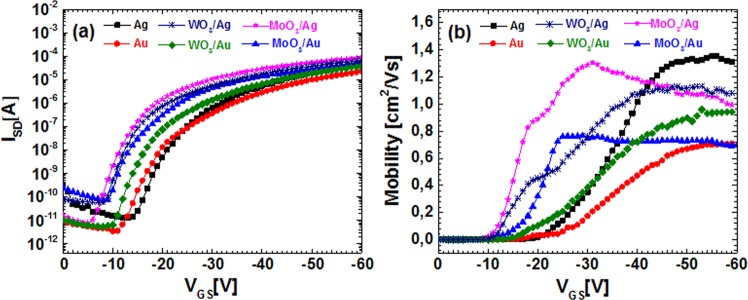
(**a**) Transfer characteristic curves in saturation regime (V_DS_ = −60 V); (**b**) gate voltage (V_GS_) dependent effective field-effect mobility of OFETs (L = 100 µm and W = 500 µm) with various source/drain electrodes.

The transfer characteristics are displayed in Fig. 3(a). Compared to the devices with bare Au and Ag electrodes, the positive shifts in threshold voltage (V_T_) are observed for all devices with WO_3_ or MoO_3_ interlayer. Specifically, the improvement in OFET performances with MoO_3_/Ag and WO_3_/Ag electrode with reference to the bare Ag electrode is more prominent than the improvement with MoO_3_/Au and WO_3_/Au electrode with reference to bare Au electrode. The key parameters, threshold voltage (V_T_), on/off current ratio and subthreshold slope (SS) were extracted from Fig. S1 according to the standard extraction method^5^, and were presented in Table 1. The devices that used MoO_3_/Ag electrodes display the best electrical characteristics with a threshold voltage of −14.7 V, an current on/off ratio of 5.1 × 10^6^, and a subthreshold slope of about 2.4 V/dec.

**Table 1:** Parameters of OFET devices with different contacts, determined from the transfer characteristics and TLM plots.

D-S electrode	µ_max_ (cm^2^/Vs)	µ_average_ (cm^2^/Vs)	V_T_ (V)	SS(V/dec)	On/Off ratio	R_C_ (kΩ.cm)
Ag	1.35	1.16 ± 0.19	−32.9 ± 3.2	3.5 ± 0.2	1.3 × 10^5^	850.5
WO_3_/Ag	1.13	1.04 ± 0.09	−17.8 ± 1.4	1.9 ± 0.1	8.2 × 10^5^	43
MoO_3_/Ag	1.30	1.12 ± 0.18	−14.7 ± 1.4	1.7 ± 0.1	5.1 × 10^6^	29.5
Au	0.69	0.61 ± 0.08	−29.4 ± 3.2	3.6 ± 0.3	2.3 × 10^6^	742
WO_3_/Au	0.96	0.86 ± 0.1	−25.1 ± 1.1	3.5 ± 0.2	3.6 × 10^6^	669
MoO_3_/Au	0.76	0.70 ± 0.06	−18.1 ± 2.1	2.8 ± 0.2	1.8 × 10^5^	120.5

Figure 3(b) shows the Field-effect mobility as a function of V_GS_ for OFET devices with various electrodes. It was calculated from the Fig. 3(a) in saturation regime based on the following equation^5^

1 $${\mu }_{sat}=\frac{2L}{W{C}_{i}}{(\frac{\partial \sqrt{{I}_{SD}}}{\partial {V}_{GS}})}^{2}$$

where *C*_*i*_ is the dielectric capacitance (total capacitance of SiO_2_ plus PVT in current work), W, L, *μ*_*sat*_, *V*_*GS*_ and *I*_*SD*_ are OFET device channel width, length, carrier mobility, gate voltage and source drain current, respectively. V_GS_ dependent mobility calculated using equation (1) gives more detailed information for further understanding the charge transfer mechanism in OFETs^14,15^. We note that the carrier field effect mobility of OFETs with various source and drain electrodes increases with increasing gate voltage, which is consistence with multiple trapping and release model in OFETs devices^14,16^. The maximum field-effect mobility (µ_max_) values in Fig. 3(b) were found to be 1.35 cm^2^/V s for bare Ag, 1.13 cm^2^/V s for WO_3_/Ag, 1.30 cm^2^/V s for MoO_3_/Ag, 0.69 cm^2^/V s for bare Au, 0.96 cm^2^/V s for WO_3_/Au, and 0.76 cm^2^/V s for MoO_3_/Au. Here, the maximum field-effect mobility is not correlated directly to the on-state current in output characteristics of OFETs. It implies that the contact resistance has less impact on effective field-effect mobility, since the mobility values are extracted in saturation regime^17^.

**Figure 4:**
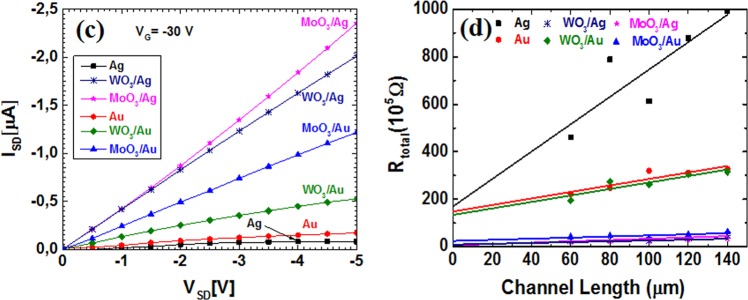
(**a**) Output curves measured at a fixed gate voltage (V_GS_ = −30 V) of OFETs devices (L = 100 µm) with different contacts (Ag, WO_3_/Ag, MoO_3_/Ag, Au, WO_3_/Au, and MoO_3_/Au). (**b**) Transmission line method (TLM) plots for various channel length devices with different contact at V_GS_ = −30 V. The intercept at L = 0 of the fitted lines determines the contact resistance (R_c_).

To quantify the impact of different interfacial layers on the performance of OFET devices, the output curves of OFETs measured at a fixed gate voltage of −30 V are shown in Fig. 4(a). The output curves in linear region directly provided the conductance G (unit in S or Ω^−1^)^18^,

2 $$G=\frac{{I}_{SD}}{{V}_{SD}}=\frac{1}{{R}_{tot}}=\frac{1}{{R}_{C}+{R}_{ch}}$$

Here, R_tot_ is the total resistance, R_ch_ is channel resistance and R_C_ is the contact resistance. As can be seen from equation (2), the slope of the curves in Fig. 4(a) related to the total resistance of an OFET device. The comparison in Fig. 4(a) shows that inserting an oxide layer such as WO_3_ or MoO_3_ between the active layer and the metal electrode results in steeper slopes than bare metal electrodes. The trend is more obvious for Ag electrode based devices. It implies that the contact configuration such as WO_3_/Ag or MoO_3_/Ag provides the more efficient exchanges of charge carriers between electrode and active layer than Au counterparts^12,19^.

According to the standard technique of transmission line method (TLM)^20,21^, we plot the total resistance (R_tot_) of the devices as a function of channel length L. The total resistance of each device is calculated from nearly linear slope in low V_SD_ region as shown in Fig. 4(a). The total resistance (*R*_*tot*_) which includes the channel resistance (*R*_*ch*_) and contact resistance (*R*_*c*_) can be obtained by derivation of drain voltage to drain current, as given by

3 $${R}_{total}=\frac{\partial {V}_{SD}}{\partial {I}_{SD}}|\begin{array}{c}{V}_{GS}\\ {V}_{SD}\to 0\end{array}={R}_{ch}+{R}_{c}$$

The channel resistance in the linear region of output curve is a function of channel length, it approximately equals to^20^

4 $${R}_{ch}=\frac{L}{W\mu {C}_{i}({V}_{GS}-{V}_{T})}$$

One can see from the equations (3) and (4), the total resistance is a linear function of channel length. Therefor, *R*_*c*_ is evaluated as *y* intercept of the linear fit of R_tot_ versus channel length, as shown in Fig. 4(b). The contact resistance at V_GS_ of −30 V is 850.5 kΩ.cm for Ag, 43 kΩ.cm for WO_3_/Ag, 29.5 kΩ.cm for MoO_3_/Ag, 742 kΩ.cm for Au, 669 kΩ.cm for WO_3_/Au, and 120.5 kΩ.cm for MoO_3_/Au electrodes, respectively. Contact resistances of the devices with MoO_3_/Ag and WO_3_/Ag electrodes are around 30 and 20 times lower than that of devices with bare Ag and Au electrode. This result is in accordance with the improvement in subthreshold region shown in Fig. 3(a), indicating that the contact resistance limits significantly the performance of OFETs devices, most notably the threshold voltage. Although the device with bare Ag electrode gives the highest mobility, as also reported in the earlier study^7^, Ag with C_8_-BTBT active layer forms highest contact resistance, and consequently, the largest threshold voltage shift.

**Figure 5:**
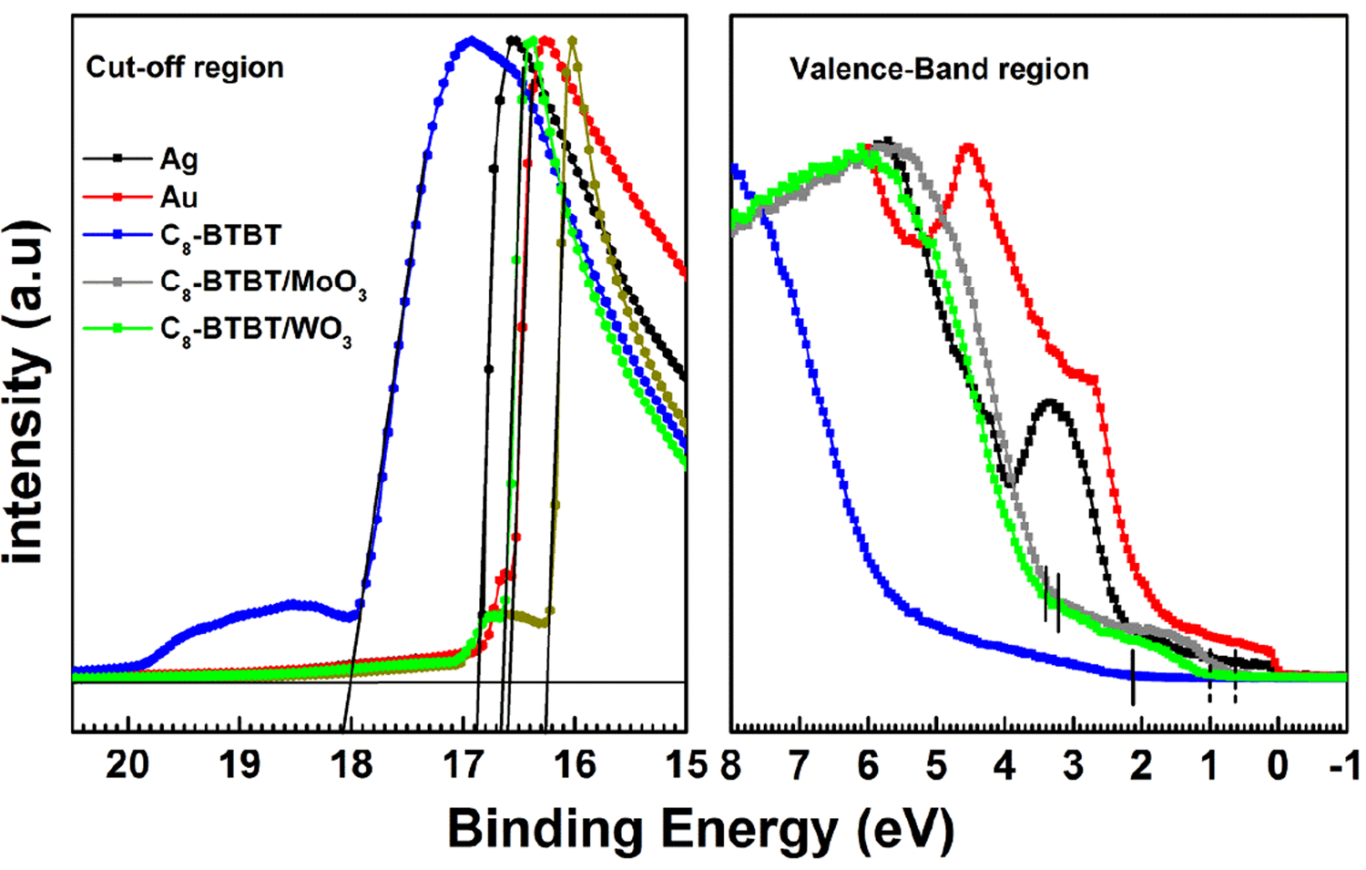
UPS spectra of Au, Ag, C_8_-BTBT, C_8_-BTBT/MoO_3_ and C_8_-BTBT/WO_3_ in secondary electron cutoff (measured with 9 V of bias) and valence region. Vertical solid lines denote the main onset of HOMO level of C_8_-BTBT and valence band maximum of the oxides. The vertical dashed lines in valence region denote the onset of gap states in MoO_3_ and WO_3_.

In order to understand the improvement in contact resistance of these devices, the electronic structures of C_8_-BTBT and various interfaces were investigated. The MoO_3_ and WO_3_ are well known transition metal oxides and widely used as interlayer between the metal electrode and organic layer in organic photovoltaic devices^22,23^. Electron affinity (EA), ionization energy (IE) and work function of transition metal oxides can be precisely measured using photoemission spectroscopy and thus very deep lying energy levels of these oxides were confirmed^24,25^. However, some recent studies still reported quite low IE for transition metal oxides^26^ or illustrated that charge transport to and from these transition metal oxides occurs via valence band maximum (VBM)^27,28^. Deep lying electronic states (position of VBM) make hole-injection very difficult through these interlayers into the organic active layer. Their interactions with different organic active layers at the interface have yet to be understood profoundly. Figure 5 shows He I UPS spectra of Ag, Au, C_8_-BTBT, MoO_3_/C_8_-BTBT and WO_3_/C_8_-BTBT films. All the spectra were normalized for visual clarity. Both secondary electron cut off and highest occupied molecular orbital (HOMO) for organic molecule (or VBM for the oxides) values were determined by linear extrapolation of the leading edge of the spectrum as shown in the figure. In the valence band region, Ag and Au show clear Fermi levels at zero binding energy. The valence band edge of C_8_-BTBT is 2.13 eV below the E_F._ With the work function which is estimated from the onset of the secondary electron cutoff, the obtained HOMO value is 5.40 eV, which is in a very good agreement with the cyclic voltammetry and optical absorption data^9^ as well as that measured by UPS^29^. Such a deep HOMO level is one of the reasons that causes large contact resistance with bare Au or Ag for C_8_-BTBT based OFETs.

**Figure 6:**
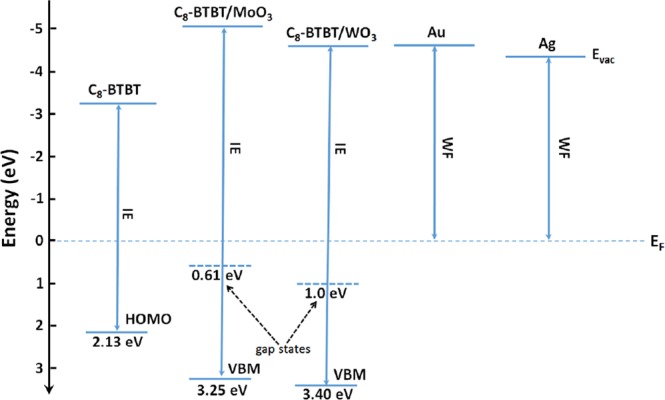
Schematic energy level diagram of C_8_-BTBT, C_8_-BTBT/MoO_3_, C_8_-BTBT/WO_3_, Au and Ag interfaces (the Fermi levels were aligned).

One can see from the right panel of Fig. 5, the valence band edges of C_8_-BTBT/MoO_3_ and C_8_-BTBT/WO_3_ are at 0.64 eV and 1.0 eV below the Fermi level, respectively (as indicated with the vertical dashed lines). If we calculate the IEs for WO_3_ and MoO_3_ using these edges and cutoff values, quite low (around 5.6 eV for both oxides) values can be obtained, which seems to contradict with those reported in literature^30^. However, when the main onsets in valence region (indicated with vertical solid lines) are taken into account as valence band edges, derived IEs are in the range of reported values. This indicates that largely delocalized gap states are formed when oxides are deposited on C_8_-BTBT, effectively moving the HOMO level of C_8_-BTBT from 2.13 eV to 0.64 eV (MoO_3_ interlayer) and 1.0 eV (WO_3_ interlayer) with respect to the fermi level, which is summarized in Fig. 6 where electronic structure of C_8_-BTBT and that of its interface with MoO_3_ and WO_3_ are presented. The Fermi levels of the layers were aligned, which led to the different vacuum levels for individual contacts at the interface. Compared to Ag and Au only electrodes, the thin MoO_3_ and WO_3_ interfacial layers on C_8_-BTBT much more reduced the injection barrier between the organic active layer and the metals, which reasonably explains the reduction in contact resistance. Even lower injection barrier with MoO_3_ interlayer also corroborates generally better performance achieved with MoO_3_ rather than with WO_3_ in OFET devices when the same metal is used.

**Figure 7:**
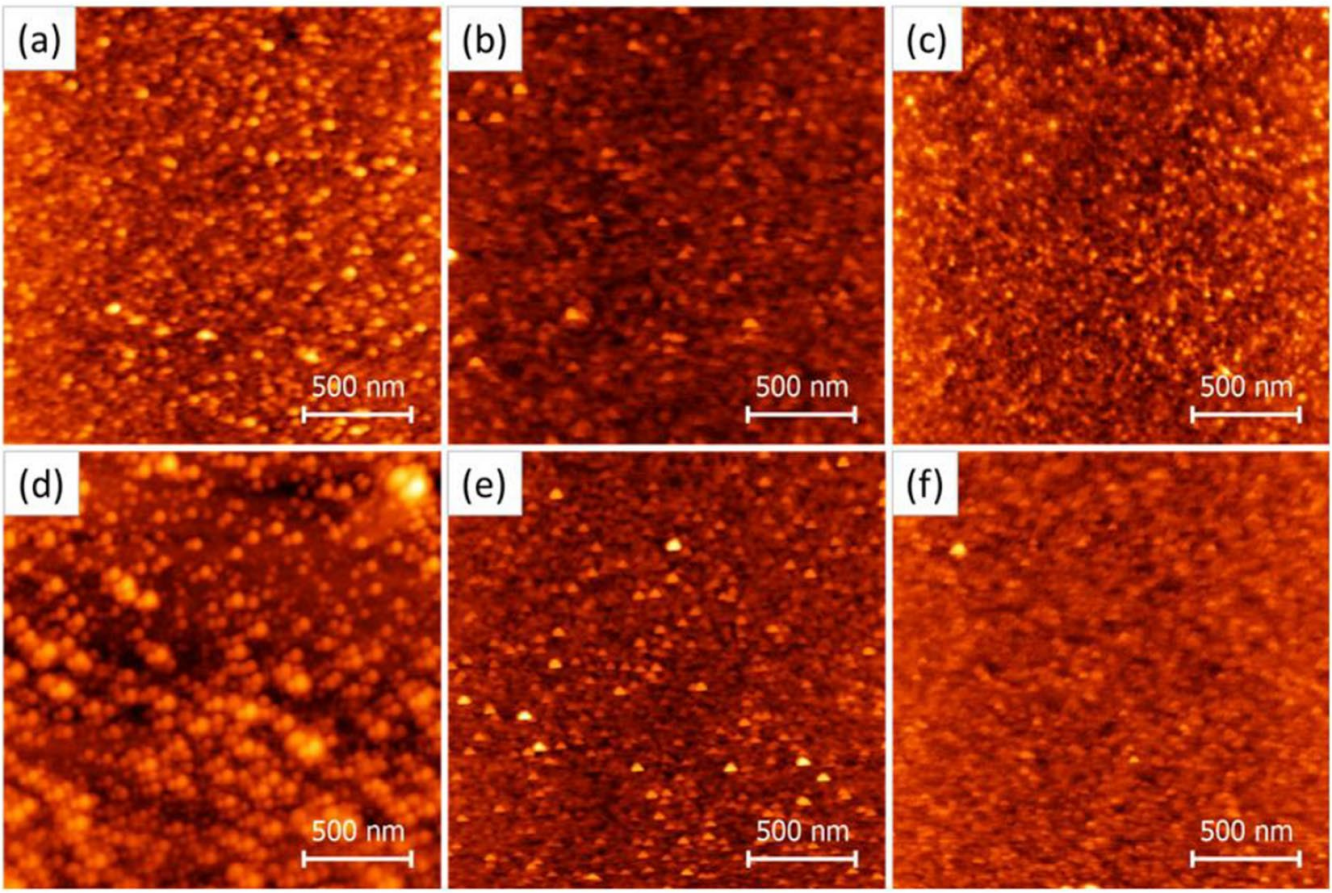
AFM height images of (**a**) MoO_3_, (**b**) Ag on MoO_3_, (**c**) Au on MoO_3_, (**d**) WO_3_, (**e**) Ag on WO_3_, and (**f**) Au on WO_3_.

However, we also note that Ag excels Au when combined with both oxide interlayers, which suggests that although the energy barrier difference at the interface is the main reason behind the difference in contact resistance, other factors, such as the morphology and incorporation of different metals into the oxide matrix also can have an impact on the performance of organic devices^31^. Morphological heterogeneity is likely to occur at different oxide/metal interfaces. AFM images of MoO_3_ and WO_3_ with and without 3 nm of Ag and Au metal layers are presented in Fig. 7. When we compare the morphologies of MoO_3_ and WO_3_ layers, the surface of the WO_3_ appeared more inhomogeneous than MoO_3_ making up of larger grains and voids. The root mean square roughness (R_MS_) of WO_3_ is 2.44 nm and higher than that of MoO_3_ (0.57 nm). Initial deposition of Ag and Au decreases both R_MS_ of MoO_3_ and WO_3_ to almost the same values (from 0.57 to 0.53 nm for MoO_3_ and from 2.44 to 0.52 nm for WO_3_), indicating that the metals diffuse and fill more voids in WO_3_ thin films compared to the MoO_3_, coming direct in contact with the active layer. Therefore, combined role of both oxide and metal can be expected at these interfaces. Considering that Ag yields higher mobility than Au, notwithstanding much larger threshold voltage shift, with the integration of MoO_3_, threshold voltage improved, at the same time, high mobility was preserved, resulting in overall best performing OFET device. These results show that although the electronic structure at organic/oxide and oxide/metal interface plays a crucial role in charge injection in OFET devices, the interface morphologies also affect the final device performances.

## Methods

In a typical procedure, C_8_-BTBT thin films were deposited by spin-coating on heavily doped silicon wafers (resistivity < 0.005 Ωcm) with thermally grown SiO_2_ (200 nm) layer. The substrates were treated by UV/ozone after usual cleaning process. Prior to C_8_-BTBT deposition, water soluble poly (1-vinyl-1, 2, 4-triazole) (PVT) was spin-coated onto SiO_2_ as passivation layer from a solvent of 3 mg/ml concentration in water, followed by an annealing process at 80 °C for two hours. C_8_-BTBT dissolved in chlorobenzene (CB) at a concentration of 10 mg/ml solution and was spin-coated (3000 rpm for 60 s) directly on top of PVT in ambient condition. For the OFETs without interfacial layer, a 60 nm gold or silver was deposited on the substrates through shadow mask using a thermal evaporator in vacuum under base pressure of 4 × 10^−6^ mbar at room temperature. For the OFETs with MoO_3_ and WO_3_ interfacial layer, 10 nm thick MoO_3_ or 10 nm thick WO_3_ was thermally evaporated at a base pressure of 4 × 10^−6^ mbar onto C_8_-BTBT active layer with a deposition rate of 0.1 nm/s through same shadow mask used for Au or Ag electrode, then Au or Ag was deposited on metal oxide layer with same thickness and procedure as mentioned above. Channel width of the devices is 1 mm, while channel lengths are varied from 60 µm to 140 µm.

The OFETs were characterized using Keithley 4200 semiconductor analyzer in a dry nitrogen glove box without exposure to air after the electrode deposition. Ultra-violate Photoelectron Spectroscopy (UPS) was done with He I (21.22 eV) photon lines from a discharge lamp. The spectrometer chamber is equipped with a SPECS PHOIBOS 100 hemispherical energy analyzer and a total energy resolution is about 140 meV for UPS as determined from the Fermi edge of clean Ag. The oxide and metal on oxide surface morphologies were investigated by atomic force microscopy (AFM) (Veeco) in tapping mode.

## Supplementary information

supplementary information (in supporting information, first and second affliations should be corrected as in the main text, thanks). 

## Data Availability

The datasets generated during and/or analyzed during the current study are available from the corresponding author on reasonable request.
