# Supplementary material for: Role of Oxide/Metal Bilayer Electrodes in Solution Processed Organic Field Effect Transistors
Source: Sci Rep. 2019 Apr 30;9:6685. doi: 10.1038/s41598-019-43237-z (PMC6491551; doi:10.1038/s41598-019-43237-z)
Supplement: Supplementary file 1 — supplementary information (in supporting information, first and second affliations should be corrected as in the main text, thanks). [file 41598_2019_43237_MOESM1_ESM.pdf]

Role of Metal oxide/Metal Bilayer Electrodes in Solution Processed Organic Field Effect Transistors

Abduleziz Ablat[1,2], Adrica Kyndiah[2], Geoffroy Houin[2], Tugbahan Yilmaz Alic[3], Lionel Hirsch[2], Mamatimin Abbas[2*]

[1]School of Physical Science and Technology, Xinjiang University, Urumqi 830046, People's Republic of China
[2]CNRS, Université Bordeaux, Laboratoire de l'Intégration du Matériau au Système (IMS), UMR 5218, ENSCBP, 16 avenue Pey Berland, 33607, Pessac Cedex, France
[3]Advanced Technology Research and Application Center, Selcuk University 42031, Campus, Selçuklu/Konya/Turkey

Supporting Information

* Corresponding authors:
E-mail Address: mamatimin.abbas@ims-bordeaux.fr (M.Abbas)

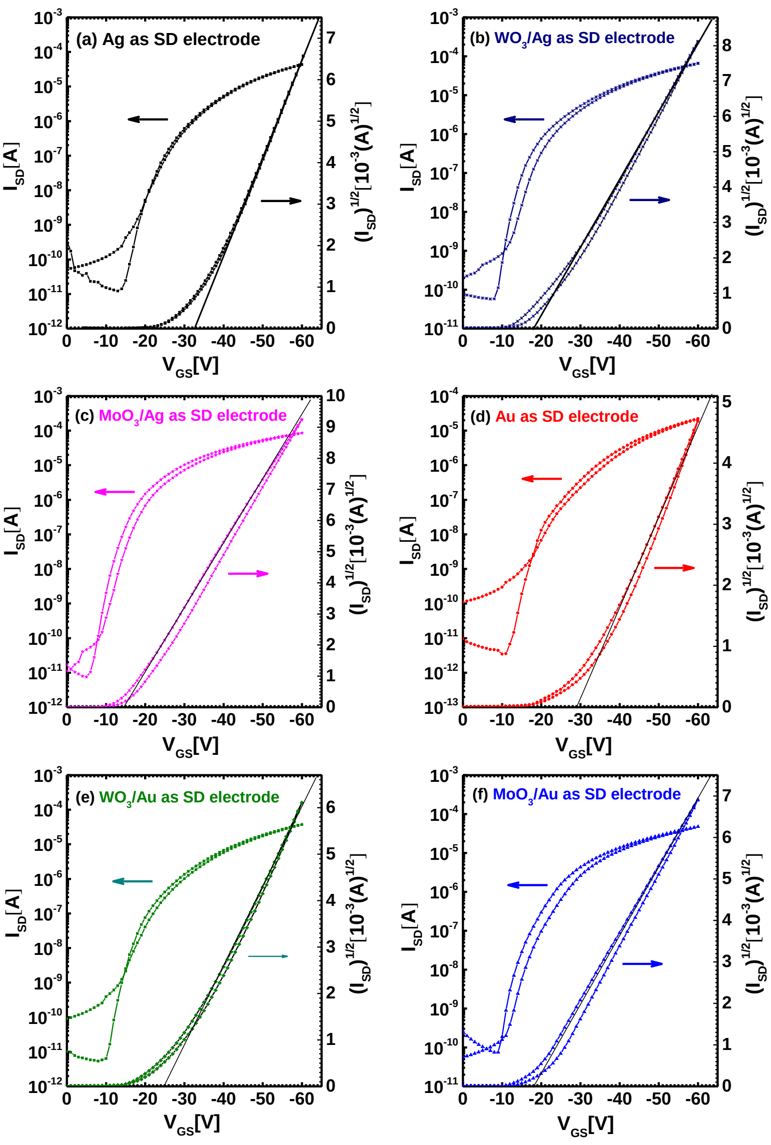

Fig .S1. A comparison of typical transfer characteristics with different source/drain electrodes of OFETs (L= 100 μm) (a) bare Ag, (b) WO_3/Ag, (c) MoO_3/Ag, (d) bare Au, (e) WO_3/Au and (f) MoO_3/Au (Forward and backward scans were recorded). Derivation of the threshold voltages are indicated by fitted solid lines.

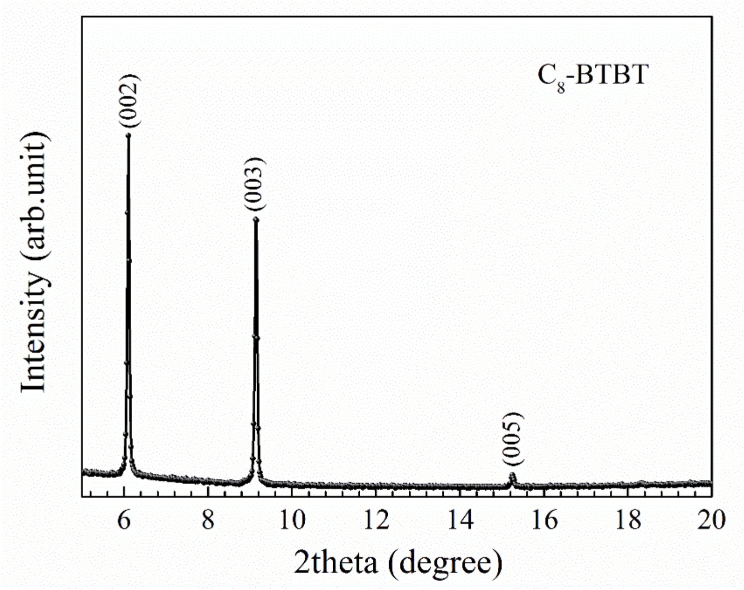

Fig .S2. XRD $\theta/2\theta$ scans of C_8-BTBT active layer in OFET devices.

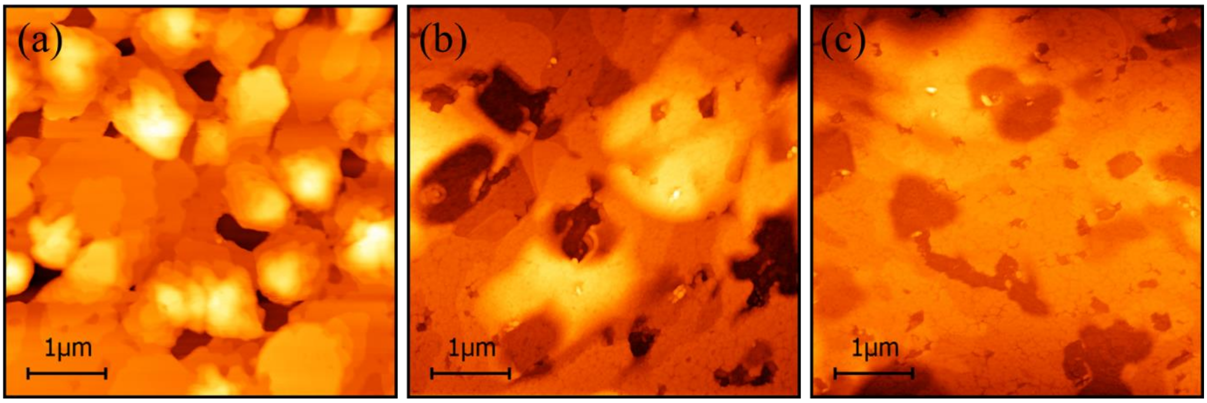

Fig .S3. AFM height images of (a) C_8-BTBT active layer, (b) Ag on C_8-BTBT, and (c) Au on C_8-BTBT.
